# A shared aperture multiport antenna for rural wireless communication and safety monitoring using TVWS, ISM, and 5G mmWave bands

**DOI:** 10.1038/s41598-025-97276-w

**Published:** 2025-04-18

**Authors:** Md Abu Sufian, Sang-Min Lee, Domin Choi, Jaemin Lee, Dongkyu Sim, Minyoung Song, Nam Kim

**Affiliations:** 1 Department of Electrical Engineering and Computer Science, Daegu Gyeongbuk Institute of Science and Technology, Daegu, 42988 South Korea; 2 Department of Corporate Support Centre, Korea National University of Transportation, Chungju, 27469 South Korea; 3 Department of Information and Communication Engineering, Chungbuk National University, Cheongju, 28644 South Korea

**Keywords:** Antenna sensor, TVWS sensing, Sub- 6 ghz and millimeter-wave sensing, Shared-aperture antenna, Multi-port antenna, MIMO communication, Safety monitoring, Disaster response, Sensor for rural areas, Electrical and electronic engineering, Techniques and instrumentation

## Abstract

To accommodate the antenna demand for rural communication and safety monitoring a shared aperture muti-port antenna sensor is presented for three different operating frequencies covering both Sub- 6 GHz and 5G millimeter-wave bands. The antenna sensor is designed on a single substrate, while different ports are connected to different radiating elements to achieve multiple frequency responses. The simulated and measured findings show that the presented antenna can cover TV-white-space (TVWS) frequency band, 5.8 GHz ISM band, and the 5G millimeter-wave frequency band. At the TVWS band, the antenna yields an omnidirectional radiation pattern with a peak gain of 3.14 dBi. While the antenna provides a unidirectional radiation pattern at the 5.8 GHz ISM and 5G millimeter-wave band with a peak gain of 6.76 dBi and 7.68 dBi, respectively. Moreover, all the antenna ports offer a radiation efficiency of more than 92%. Additionally, the 2-port MIMO configuration at the 5G millimeter-wave band shows excellent MIMO diversity performances by utilizing the proposed novel decoupling structure, which consists of metallic stub and cavity vias. Overall performance of the proposed antenna, especially the three operating frequency band including the TVWS band, makes it a viable solution for the sensing and communication in rural areas.

## Introduction

In recent years, with the advancement of wireless communication technologies, antennas offering multiple frequency bands are becoming exceptionally popular among researchers for their ability to perform the tasks of multiple antennas^1,2^. These antennas are vital for resolving the various challenges of rural sensing applications including monitoring environmental conditions and agricultural activities, seamless wireless connectivity, and efficient disaster management^3^. However, selecting the antenna operating frequency for rural sensing and communication applications is very critical. As there is an antenna design tradeoff between the operating frequency and the coverage area. The lower frequency band outperforms the higher frequency band in terms of the antenna coverage area. While the higher frequency band such as the 5G millimeter-wave band offers a wide range of antenna bandwidth to accommodate a vast number of users with ultra-fast data transfer capabilities^4,5^.

Recently, the TV white space (TVWS) frequency bands, which refer to the unused frequency spectrum of television broadcasts (between 470 MHz and 780 MHz), have become very popular among researchers for their benefit of covering longer distances^6,7^. TVWS can be an excellent solution to communicate in rural and remote areas using TV broadcast towers where the number of base stations is limited. However, very few printed TVWS antennas have been reported in the literature^7–9^. In^8^, a system-embedded TVWS antenna is presented. The antenna offers a good omnidirectional radiation pattern; however, the antenna suffers from a low gain and does not cover the whole TVWS frequency band. On the other hand, the antennas in^8^and^9^provide a good gain but they occupy a very large antenna area^7^or cannot offer the full TVWS frequency band^9^.

Additionally, the 5.8 GHz ISM band and the 5G millimeter-wave frequency band are adopted rapidly in modern wireless communication modules for short-distance body area networks^10,11^and ultra-fast communication^12,13^, respectively. Several antennas are reported in the literature for the 5.8 GHz band^10,11,14–16^and 5G millimeter-wave frequency band^12,13,17–20^applications. In these works^10–20^various techniques have been utilized to improve the antenna performances in terms of bandwidth, gain, and radiation efficiencies. However, none of these works offer the TVWS frequency band along with the 5.8 GHz ISM or the 5G millimeter-wave band. To achieve the multi-standard operating frequency band from a single antenna module, the shared aperture antenna techniques can be utilized^20–26^. A beam-steerable shared aperture antenna is presented in^20^for 3.5 GHz and 28 GHz dual-band applications. Where multiple substrate and metallic layers along with metallic vias are utilized to achieve beam-steerable and dual operating frequency capabilities. In^21^electromagnetic structures are adopted to create the shared aperture antenna for four different frequency bands. Similarly, the others reported works^22–26^used multiple substrate layers with different techniques to create the multiband shared aperture antenna, Fabry-Perot cavity with folded reflect array^22^, magnetoelectric dipole^23^, artificial magnetic conductor^24^, quasi-Yagi structure^25^, and metasurface structure^26^. However, none of these reported works offers the TVWS operating band. And all of these works suffer from complex design procedures.

**Fig. 1:**
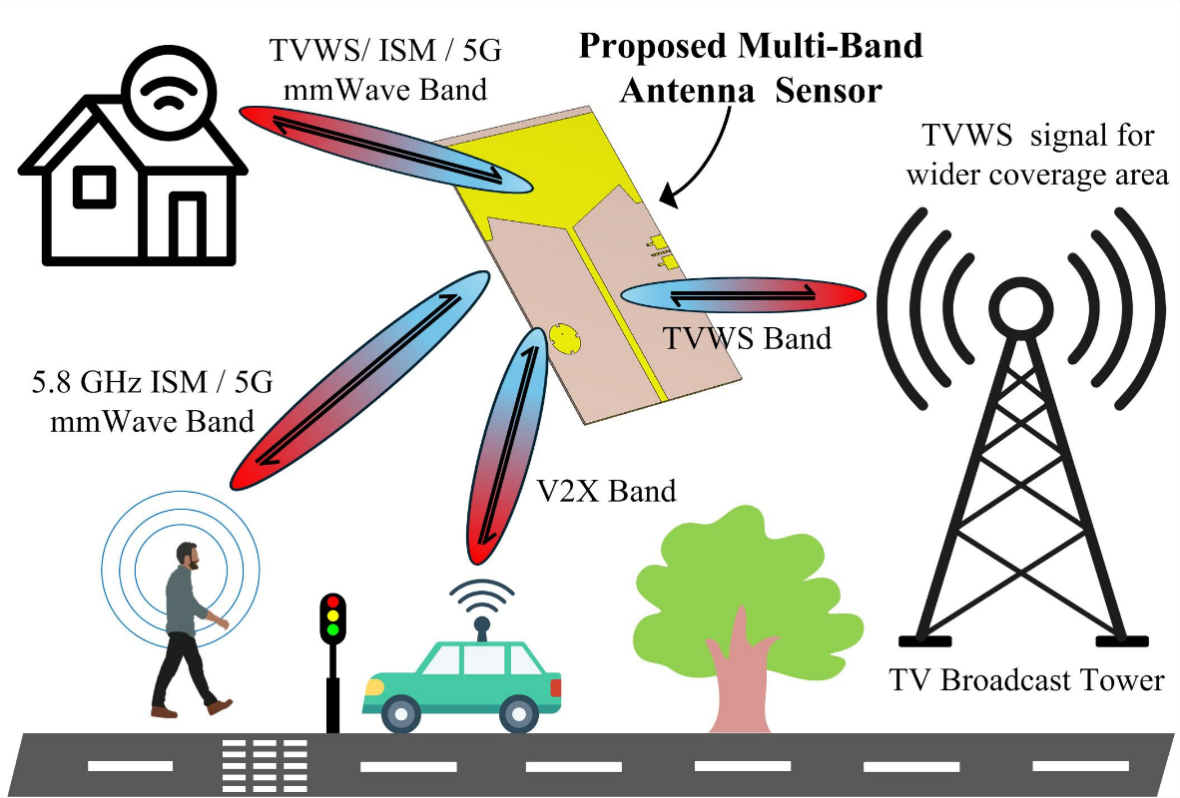
The communication and sensing scenario of the proposed shared aperture multi-band antenna.

In this proposed work a shared aperture multi-port antenna sensor is presented for three different operating frequencies. The communication scenario of the proposed shared aperture multi-band antenna is presented in Fig. 1. Initially, a TVWS antenna is designed with an omnidirectional radiation pattern to sense any signal from any distant metropolitan area. The TVWS antenna consists of a slotted monopole that utilizes a partial ground plane.

Subsequently, to fully reuse the antenna substrate a circular antenna with four rectangular slots is designed and optimized at the lower left side of the feedline of the TVWS antenna for the 5.8 GHz ISM band. Afterward, on the right side of the substrate, a 2-port 5G millimeter-wave MIMO antenna is created for the wideband 5G communication, while a novel decoupling technique consisting of parasitic element with cavity vias is proposed and utilized efficiently to achieve high isolation between the MIMO elements. To the author’s knowledge, no antenna modules have been reported in the literature that operate at the TVWS band while also covering the 5.8 GHz ISM band and the 5G millimeter-wave band. The proposed design approach enables the antenna system to sense and communicate efficiently at both shorter and longer distances due to its combination of very low and high operating frequencies. The CST 2023 academic software is used for all the antenna simulations^27^. Then, to validate the proposed work a prototype of the proposed antenna is manufactured and tested. The rest of the paper is organized as follows. The antenna geometry and design procedure are presented in section II. Whereas section III contains the results and discussion. Section IV presents a performance comparison with the other documented shared-aperture antennas in the literature. Finally, section V conveys the conclusion of the proposed work.

## Proposed shared-aperture multi-port antenna design

This section explains the method by which the proposed antenna is designed. The shape and other design parameters of the antenna are provided in the first subsection. And in the following subsection the design process is described.

### Geometry of the proposed antenna sensor

**Fig. 2:**
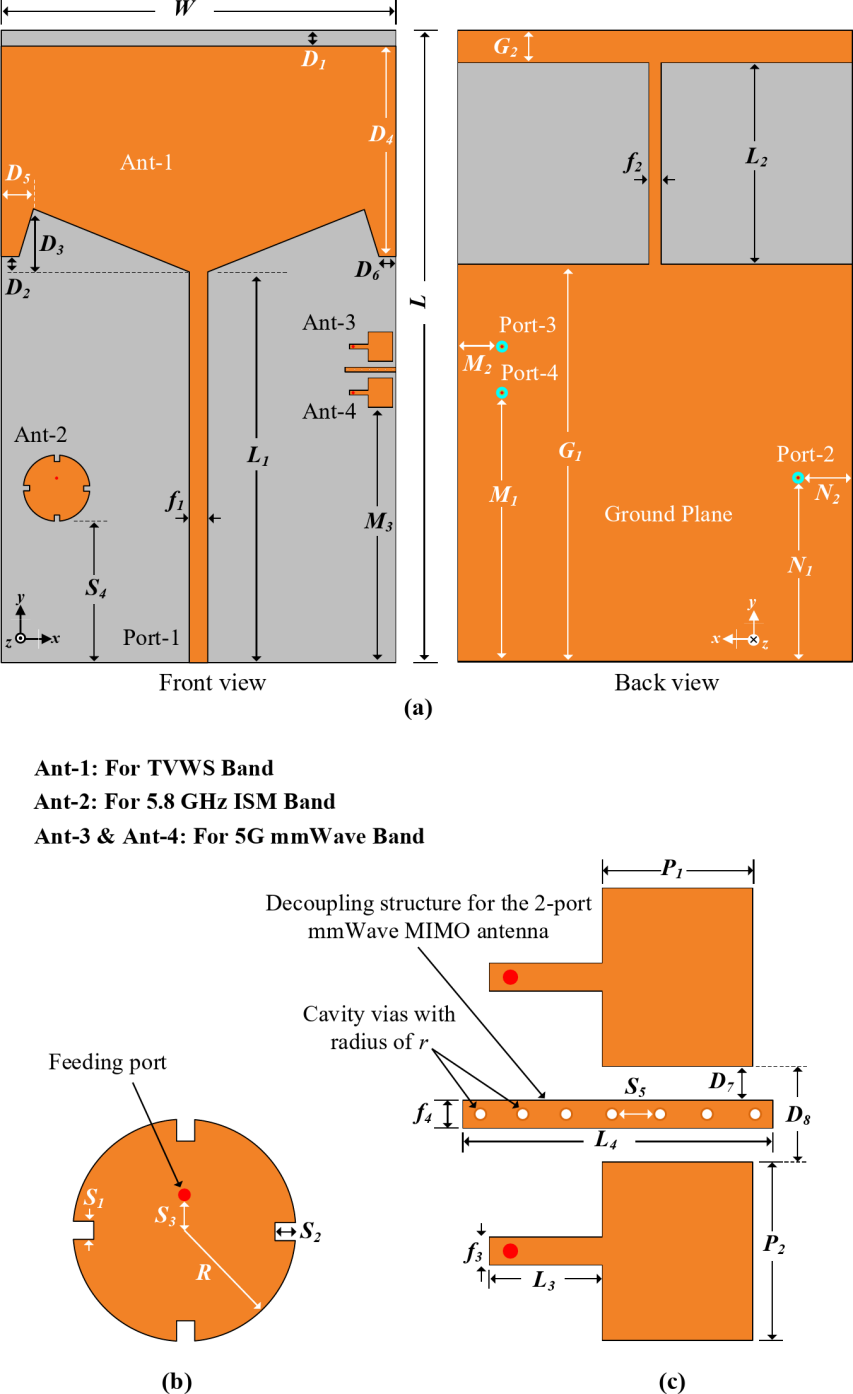
Schematic and design parameters of the proposed shared-aperture multi-port antenna sensor (**a**) front and back view (**b**) design parameters of Ant- 2, and (**c**) design parameters of Ant- 3 and Ant- 4.

The layout of the proposed shared-aperture multi-port antenna sensor for TVWS, 5.8 GHz ISM and 5G millimeter-wave frequency band is depicted in Fig. 2. The proposed antenna sensor is created on a 1.52 mm thick Taconic TLY- 5 substrate having a relative permittivity of 2.2 and loss tangent of 0.0009. In the following subsection, the antenna design strategy is explained. And the optimized values of the design parameters for the proposed antenna are listed in Table 1.

### Design procedure

A detailed step-by-step design procedure of the proposed shared-aperture multi-port antenna sensor is conveyed in this sub-section along with the results of different design stages. This sub-section also explained the critical design parameters which help the antenna optimization to achieve the targeted performance.

#### TVWS antenna design for wider sensing coverage area

**Fig. 3:**
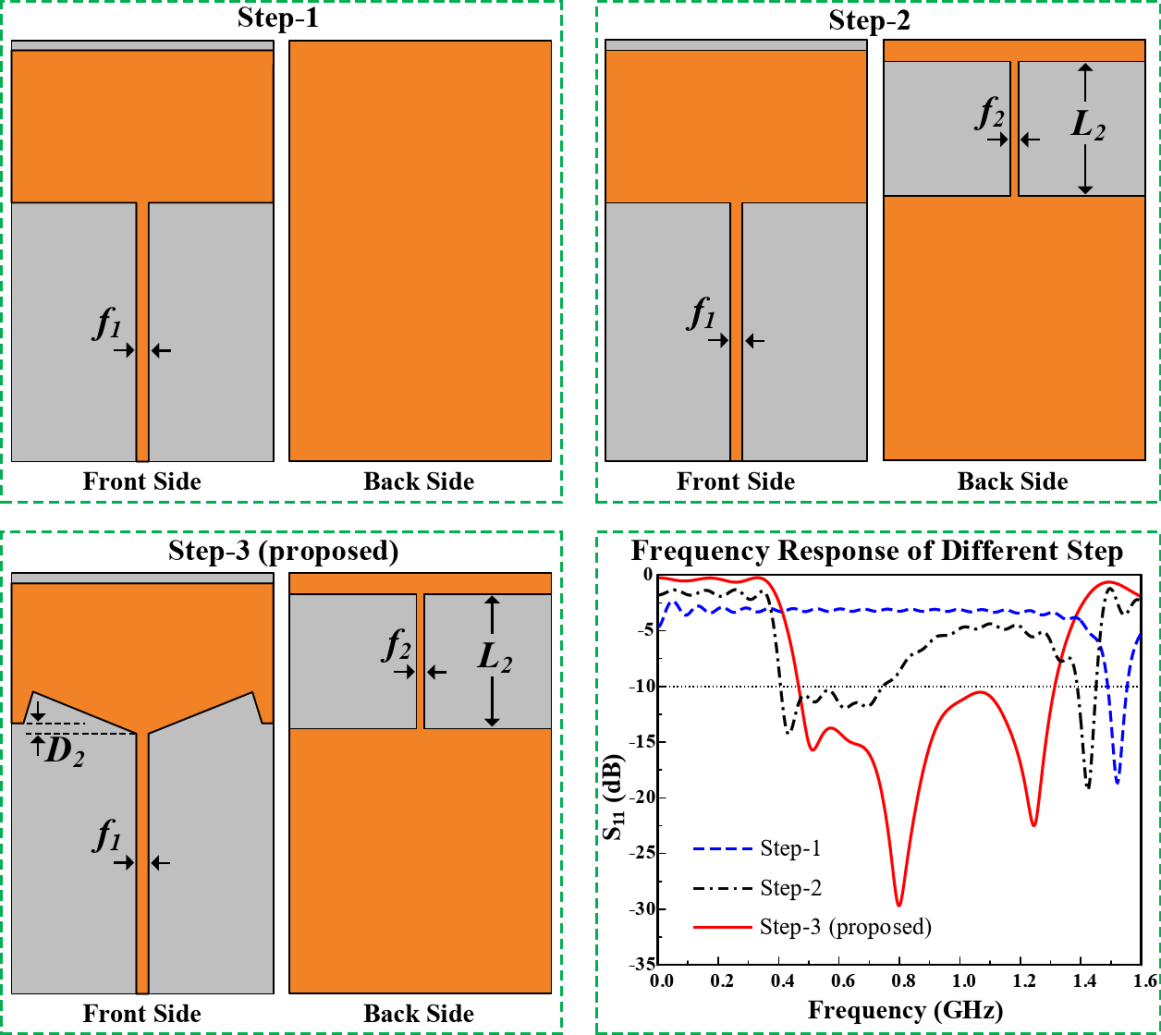
Different design stages for the initial TVWS band antenna and its frequency responses.

The design process of the proposed shared-aperture multi-port antenna sensor is started by designing a monopole antenna on a 1.52 mm thick Taconic substrate for the TVWS frequency band, while the edge feeding technique is.

utilized. Major design stages and the corresponding frequency responses of the TVWS band are presented in Fig. 3. The initial width (W) of the radiating element is predicted using the Eq. (1)^28^, whereas *c* is the speed of light, *f*_*r*_ is the targeted frequency, and $\varepsilon_{r}$ is the substrate’s dielectric constant. Afterwards the parameters are optimized for better performance.

1 $$\:W=\frac{c}{2{f}_{r}}\sqrt{\frac{2}{{\varepsilon}_{r}+1}}$$

**Table 1:** Optimized values of design parameters (unit = millimeter).

Variable	Value	Variable	Value	Variable	Value	Variable	Value
*W*	111	*G*_*1*_	111.8	*L*_*3*_	5	*P*_*2*_	8
*L*	178	*G*_*2*_	8.9	*L*_*4*_	13.5	*R*	9.35
*D*_*1*_ *= D*_*2*_	4.44	*f*_*1*_	5	*S*_*1*_	1.75	*M*_*1*_	76
*D*_*3*_	17.8	*f*_*2*_	3.11	*S*_*2*_	1.7	*M*_*2*_	12
*D*_*4*_	59.12	*f*_*3*_	1.2	*S*_*3*_	3	*M*_*3*_	72
*D*_*5*_	9	*f*_*4*_	1	*S*_*4*_	44	*N*_*1*_	51.65
*D*_*6*_ *= D*_*8*_	5	*L*_*1*_	110	*S*_*5*_	1.47	*N*_*2*_	15.5
*D*_*7*_	2	*L*_*2*_	57.3	*P*_*1*_	7	*r*	0.3

It can be observed from Fig. 3 that, initially the designed antenna, step- 1, offers a very narrow bandwidth. And step- 2 doesn’t offer a good frequency response. While the proposed structure, triangular slot on the radiating patch and square slot on the ground plane, as presented in step- 3 offers a very good frequency response with a wide operating bandwidth. All the design parameters are depicted in Fig. 2, and the optimized values are listed in Table I.

**Fig. 4:**
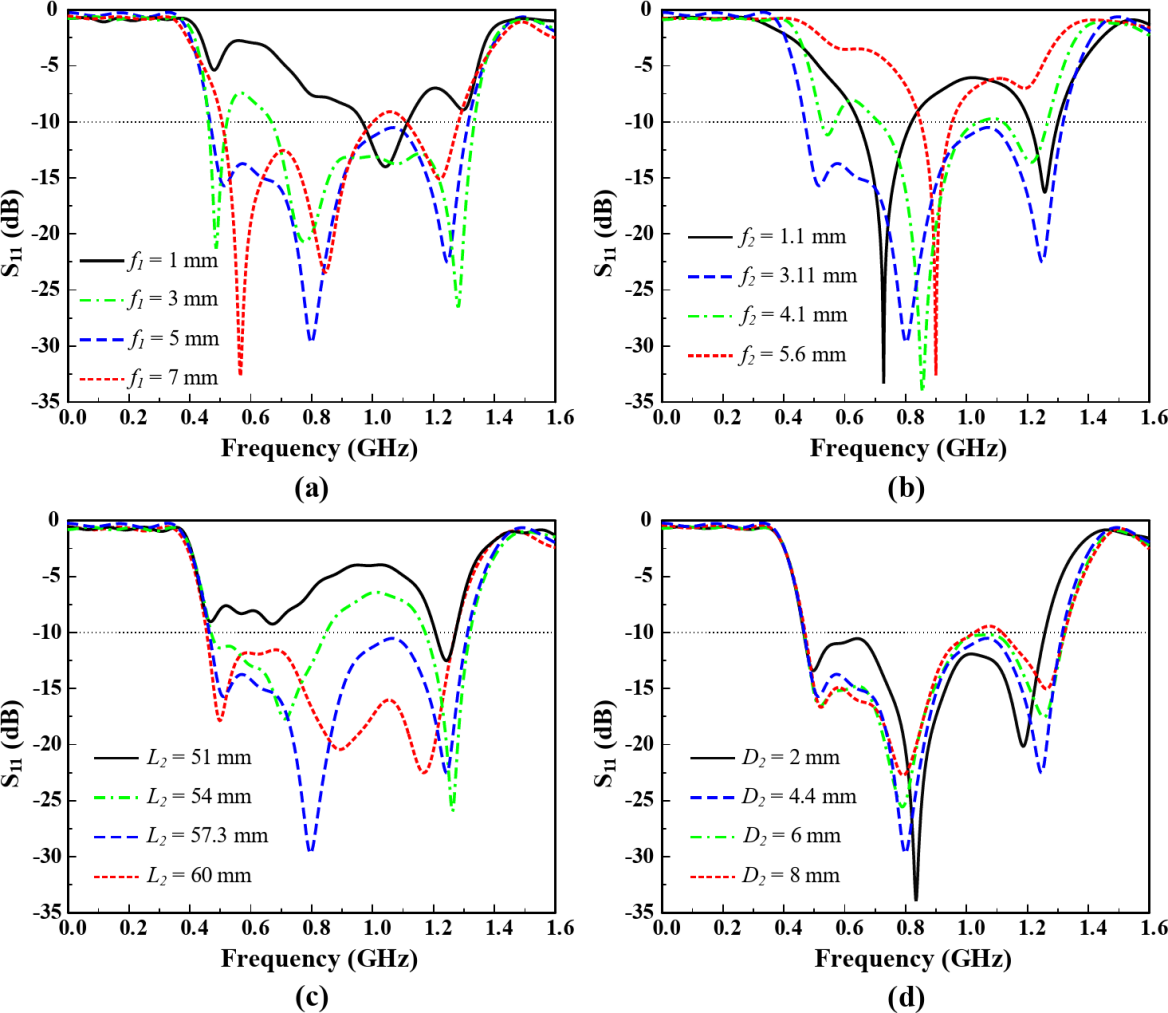
Parametric study of Ant- 1 for the TVWS band, (**a**) *f*_*1*_, (**b**) *f*_*2*_, (**c**) *L*_*2*_, and (**d**) *D*_*2*_.

Figure 4 conveyed the parametric study of the TVWS band antenna for four different paraments. These parameters has the significant effect on the frequency response than the other parameters. However, the final results are achieved by optimizing all the parameters. It can be observed from Fig. 4 that the antenna frequency can be adjusted based on the requirements by controlling the proposed design parameters.

**Fig. 5:**
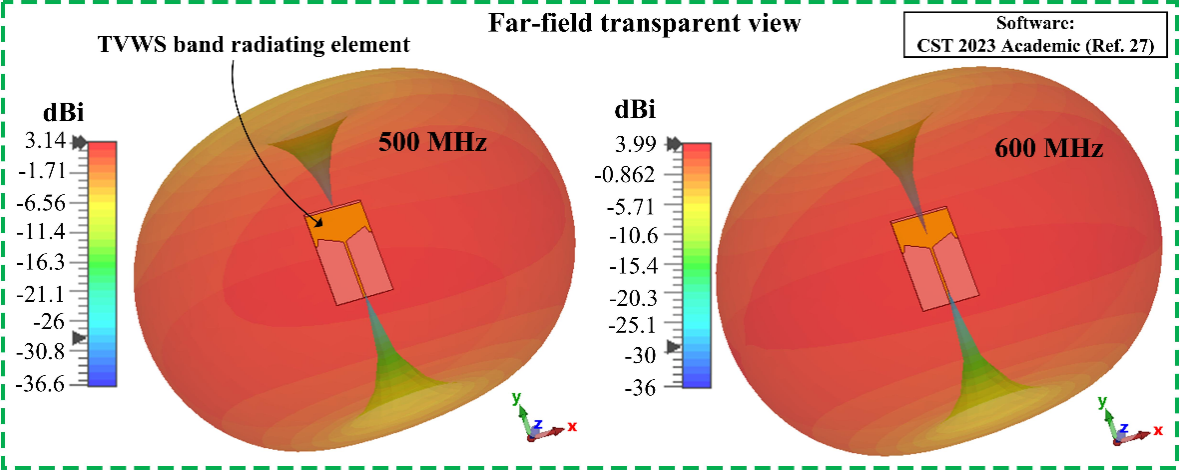
3D radiation behavior of the designed TVWS band element at 500 MHz and 600 MHz.

3D radiation pattern of the designed TVWS band antenna is illustrated in Fig. 5 at 0.5 GHz and 0.6 GHz. The antenna offers an ideal omnidirectional radiation pattern in the operating band. At the TVWS band the antenna offers a gain of more than 3.14 dBi within the functional frequency band, with a radiation efficiency of 92%. Deu to the omnidirectional radiation characteristics the designed TVWS antenna can act as an antenna sensor which will collect data from all directions. Additionally, the benefit of this TVWS band is that the signal can be sent from very long distances. Therefore, this antenna sensor will benefit rural communication where the numbers of base station are limited.

#### 5.8 GHz band circular patch design

After getting good results for the TVWS band, a circular radiating element is designed on the same substrate for the 5.8 GHz ISM band as shown in Fig. 2. The idea is to fully utilize the substrate aperture and achieve multiple standard frequency bands. The radius *R* of the circular patch is calculated using Eq. (2) and Eq. (3)^28^. Here, the *h* is the substrate height, $\varepsilon_{r}$ represent the substrate’s dielectric constant, and the targeted frequency is *f*_*r*_.

**Fig. 6:**
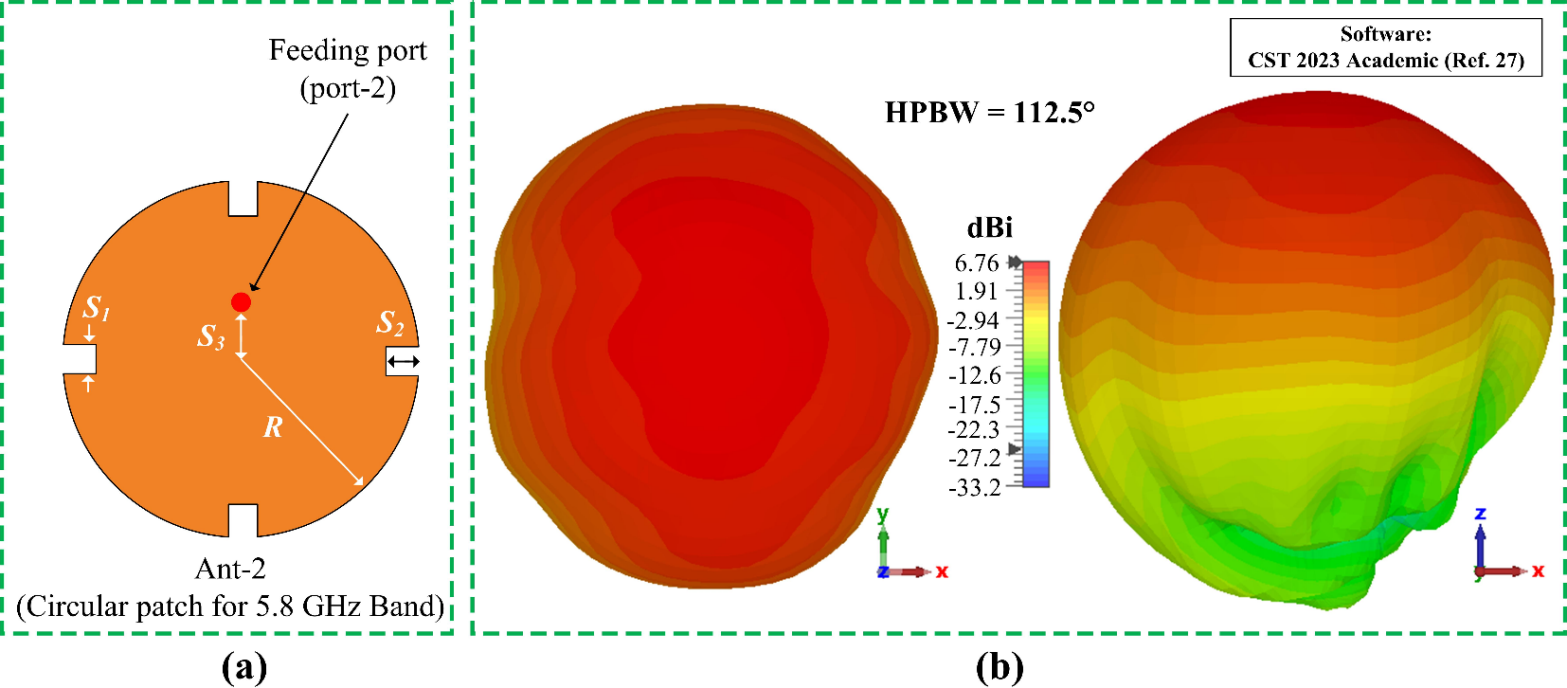
Design and performance of the circular patch (**a**) schematic layout, and (**b**) 3D radiation pattern with gain value at 5.8 GHz.

Subsequently the antenna radius *R* along with the feed position *S*_*3*_ from the center is optimized to achieve the optimum performance. Additionally, four rectangular slots are created on the designed circular patch, which offers a little improvement of the antenna directivity. The proposed circular patch and its 3D radiation performance at 5.8 GHz is illustrated in Fig. 6. Although the designed circular patch offers a unidirectional radiation pattern, it provides a wide coverage angel by having a half-power-beam-width (HPBW) of 112.5° and a radiation efficiency of 94%. Due to the wide coverage angel with high gain, this circular patch can be used as an efficient sensor for the 5.8 GHz band sensing and communications.

2 $$\:R=\:\frac{F}{{\left\{1+\frac{2 h}{\pi\:{\varepsilon}_{r}F}\left[\text{ln}\left(\frac{\pi\:F}{2 h}\right)+1.7726\right]\right\}}^{1/2}}$$

3 $$\:F=\:\frac{8.791\times\:{10}^{9}}{{f}_{r}\sqrt{{\varepsilon}_{r}}}$$

**Fig. 7:**
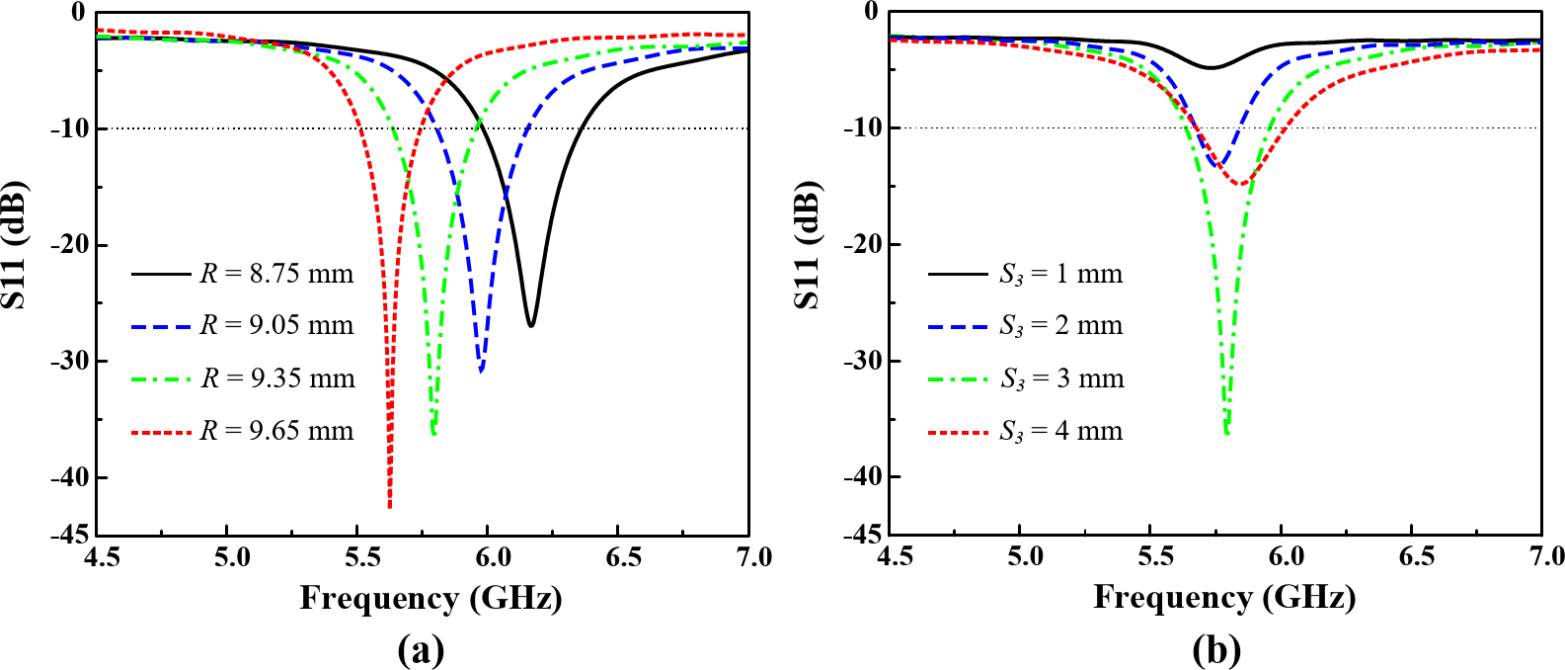
Parametric study and frequency response of Ant- 2 for the 5.8 GHz band for different parameters (**a**) *R*, and (**b**) *S*_*3*_.

The frequency response of this circular patch is mostly dependent on the parameter *R* and *S*_*3*_ as shown in Fig. 7. It can be observed from Fig. 7 (a) that the resonant center frequency of the circular patch can be controlled by optimizing the radius *R* of the circular patch. By increasing the value of *R*, the center frequency shifts from upper band to the lower band. Similarly, by decreasing the values of *R* the circular patch can be tuned for the upper frequency band. Additionally, by controlling parameter *S*_*3*_, which represents the position of the feeding of the circular patch from the center, performance of the resonance can be improved. The parametric study for *S*_*3*_ is presented in Fig. 7 (b). The finalized parameter values of this circular patch are listed above in Table I. The proposed design of this circular patch yields an operating bandwidth of 330 MHz covering both 5.8 GHz ISM band and the 5.9 GHz vehicle to everything (V2X) band.

#### Wideband 5G Millimeter-Wave 2-Port MIMO design

**Fig. 8:**
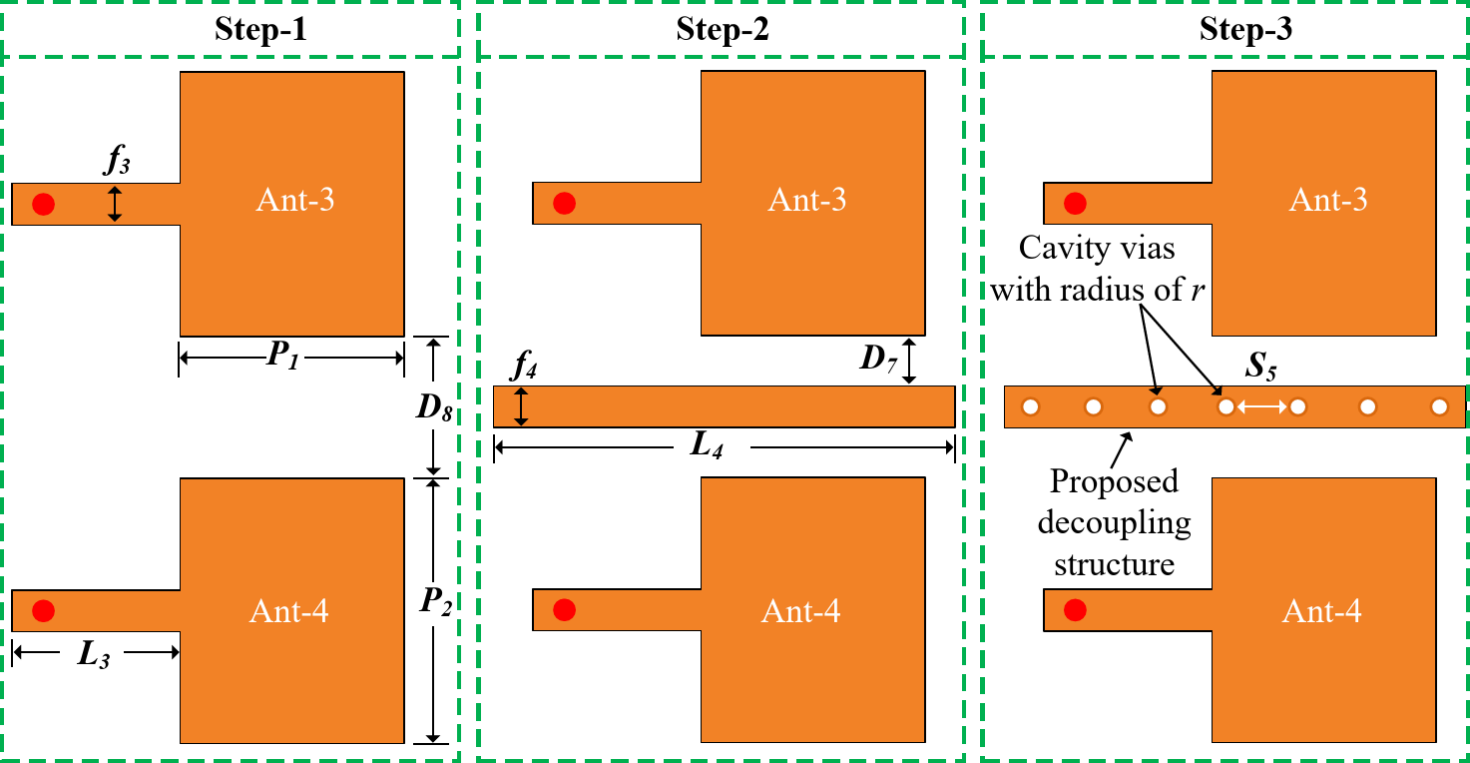
Different design stages of the proposed decoupling structure of the 2-port MIMO elements for the 5G millimeter-wave band.

To support the 5G millimeter-wave communication, a 2-port multiple-input multiple-output (MIMO) configuration is designed on the right side of the substrate as shown in Fig. 2. Initially Ant- 3 is designed for a wideband operating frequency from 23.02 GHz to 30.37 GHz. While Eq. (1) is utilized to obtain the width of the Ant- 3. After satisfying the performance of Ant- 3 an identical structure is created very near to Ant- 3 which is denoted as Ant- 4. The distance between Ant- 3 and Ant- 4 is 5 mm. In this work we kept the Ant- 3 and Ant- 4 as close as possible to show that many more millimeter-wave elements can be integrated on the right side of the substrate. To maintain high MIMO isolation with lower element spacing, a novel decoupling structure, parasitic stub with cavity vias is proposed and implemented. The design stages of the proposed decoupling structure are conveyed in Fig. 8.

**Fig. 9:**
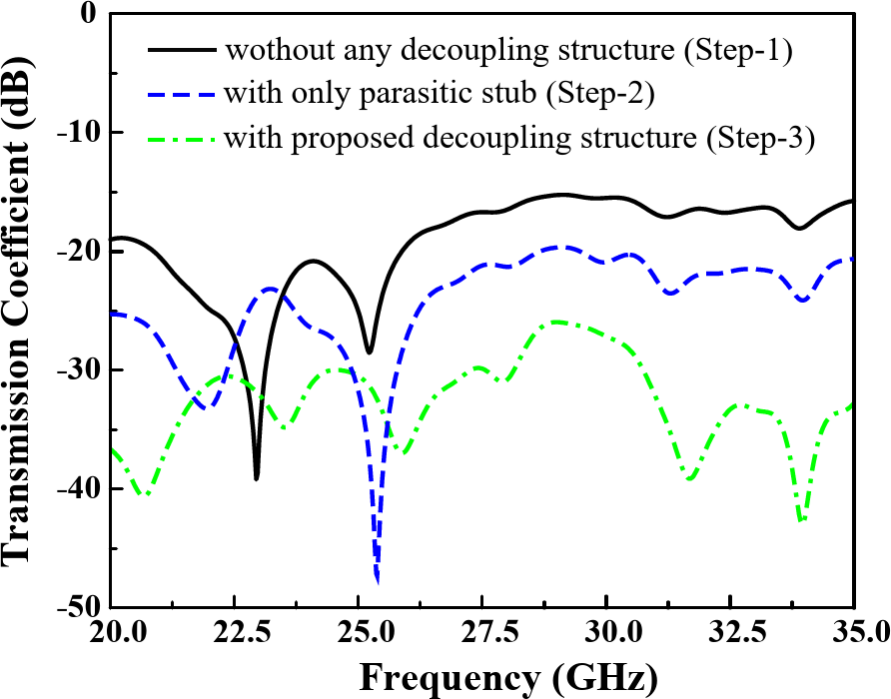
Transmission coefficient of the 2-port MIMO elements for the different decoupling structures.

The transmission coefficient of the 2-port 5G MIMO for the different design stages is presented in Fig. 9. The others performance parameters including reflection coefficient, radiation pattern, and gain of the proposed 2-port MIMO elements are discussed later in the Antenna Results section. Here only the transmission coefficient and surface current are studied to show the effectiveness of the proposed decoupling structure. It can be observed from the transmission coefficient of different design stages of the decoupling structure in Fig. 9 that the proposed decoupling structure offers a significant isolation between the MIMO elements. Absolute value of the transmission coefficient is referring to the isolation between the MIMO elements. Without the decoupling structure as in Step- 1, the MIMO elements show a low minimum isolation of around 16 dB within the functional band. While with the parasitic element an improved minimum isolation of 19 dB is obtained. And with the proposed decoupling structure, parasitic stub with cavity vias, offers a very high minimum isolation of more than 26.4 dB within the operating frequency band.

**Fig. 10:**
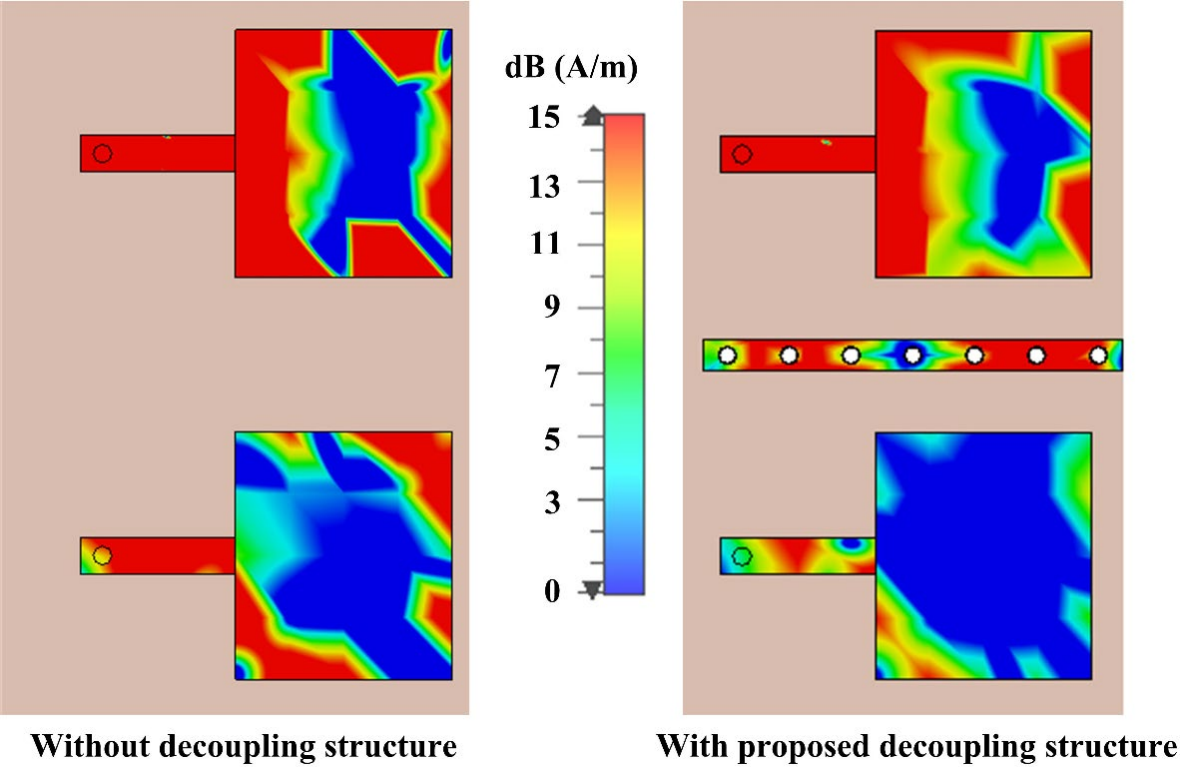
Surface current behavior of the 2-port MIMO elements at 28 GHz with and without the decoupling structure while Ant- 3 is excited.

The surface current of the MIMO elements for with and without the proposed decoupling structures is studied and displayed in Fig. 10 to show the effectiveness of the proposed isolation enhancement method, while port of Ant- 3 is excited. Without the decoupling structure the Ant- 4 is affected by the current of Ant- 3. On the other hand, with the proposed decoupling structure, parasitic stub with cavity vias, the Ant- 4 shows very negligible influence due to the excitation of Ant- 3. Thus, a high isolation between the MIMO elements is achieved.

#### Mutual coupling between all ports

**Fig. 11:**
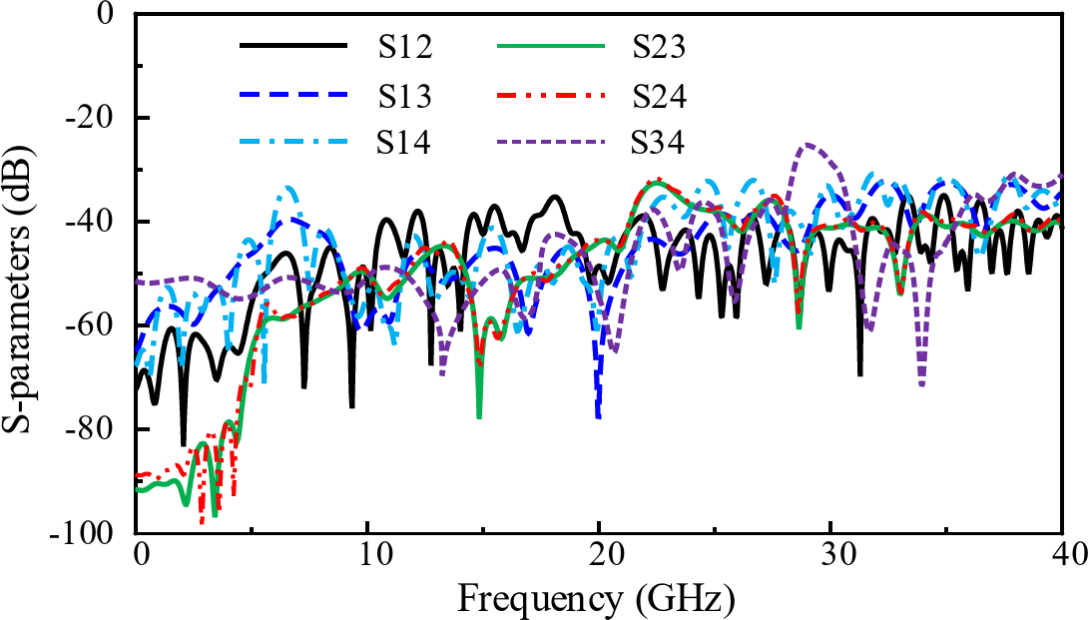
Mutual coupling between all antenna ports; Ant- 1, Ant- 2, Ant- 3, and Ant- 4.

The mutual coupling of all ports of the proposed shared-aperture antenna is presented in Fig. 11. The absolute value of the mutual coupling also refers as the port isolation. It can be seen that the coupling between the Ant- 3 and Ant- 4 is the maximum among the antenna ports. Without the proposed decoupling structure between Ant- 3 and Ant- 4, the coupling was too high which is shown earlier in Fig. 9. It can be observed from the Fig. 11 that the isolation among all the others ports are less than 32 dB.

## Performance of the proposed antenna sensor

**Fig. 12:**
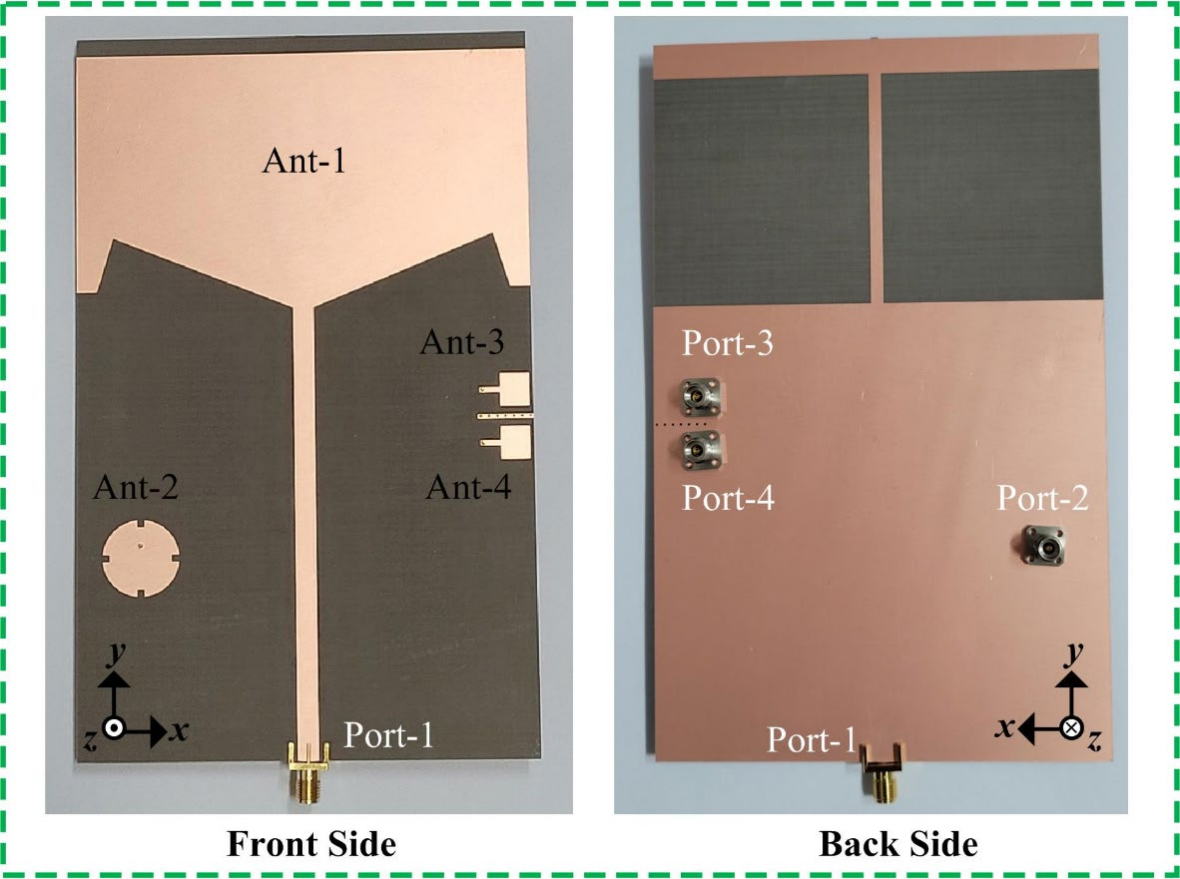
Prototype of the proposed shared-aperture multi-port antenna sensor.

The finalized simulated and measured results of the proposed shared-aperture multi-port antenna sensor are presented in this section. While the prototype of the proposed shared-aperture multi-port antenna sensor is displayed in Fig. 12. All the S-parameters are measured using Keysight frequency measurement equipment in open environments. While the far-field characterization is done by a commercial company in anechoic chambers^29^. For Ant- 1 and Ant- 2 in low frequency chamber, while the radiation behavior of Ant- 3 and Ant- 4 is characterized in a millimeter-wave high frequency chamber.

### Characteristic analysis of the Ant- 1

#### Reflection coefficient

**Fig. 13:**
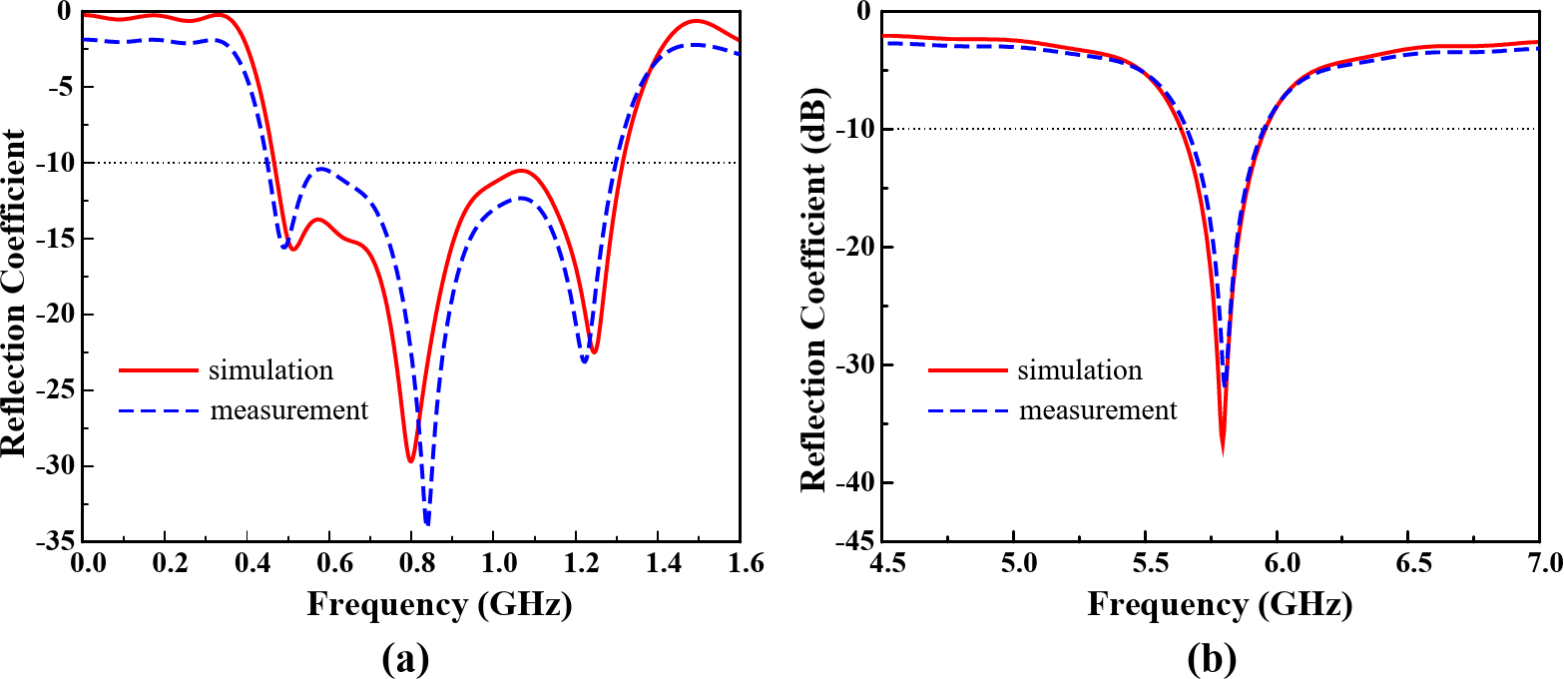
Frequency response of Ant- 1 and Ant- 2 for both simulation and measurement (**a**) Ant- 1, and (**b**) Ant- 2.

The simulated and measured frequency response of the Ant- 1 of the proposed shared-aperture multi-port antenna sensor in terms of reflection coefficient is presented in Fig. 13(a), while Fig. 13(b) contains the reflection coefficient of Ant- 2. It can be observed from Fig. 13 (a) that the Ant- 1 offers a very wide operating bandwidth (S_11_<–10 dB) of more than 840 MHz (from 0.467 GHz to 1.31 GHz). Which covers the entire TVWS band (between 470 MHz and 780 MHz). Additionally, the Ant- 1 also covers the L2 (1227.6 MHz), L5 (1176 MHz) GPS satellite bands, and 1.2 GHz satellite band. The operating frequency of Ant- 1 makes it a good fit to perform as an antenna sensor to sense multiple broadcast signal including, TVWS, GPS and satellite signals.

#### Radiation behavior and sensing capabilities of Ant- 1

**Fig. 14:**
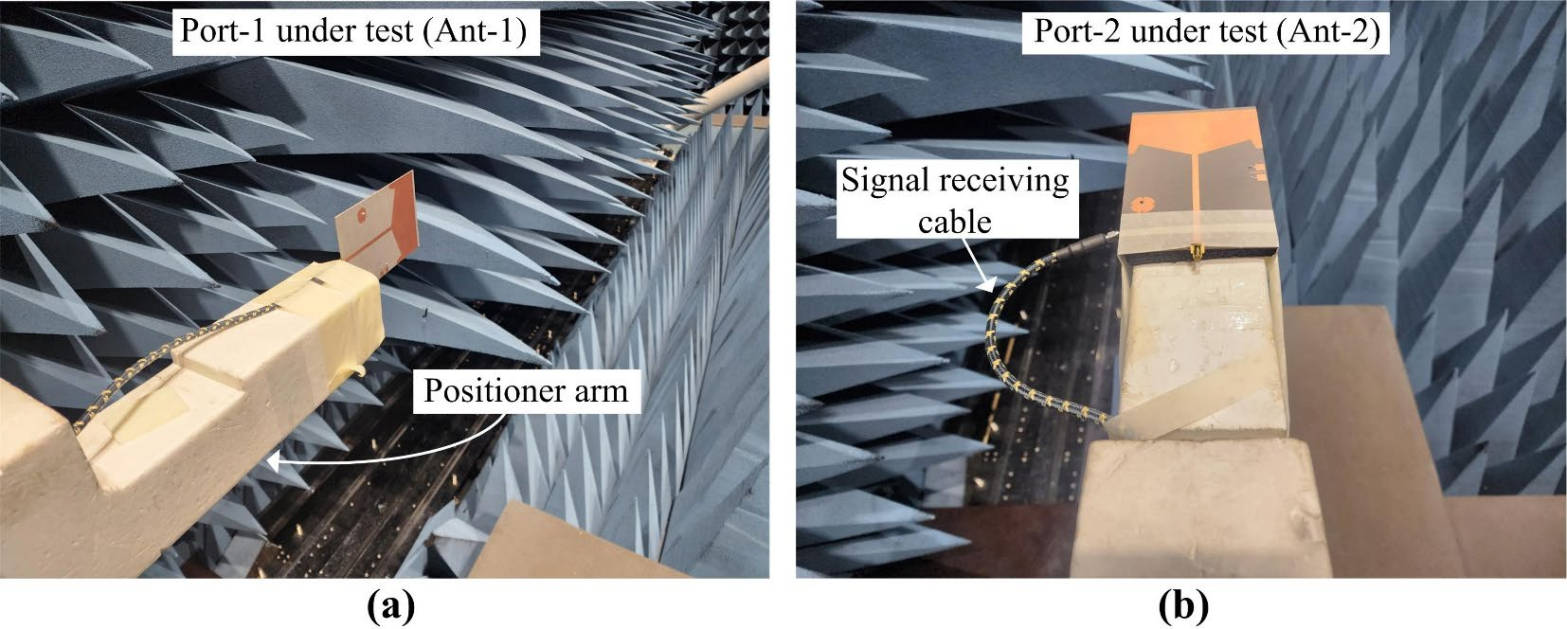
Radiation measurement setup for different antenna ports in the anechoic chamber (**a**) port- 1, and (**b**) port- 2.

**Fig. 15:**
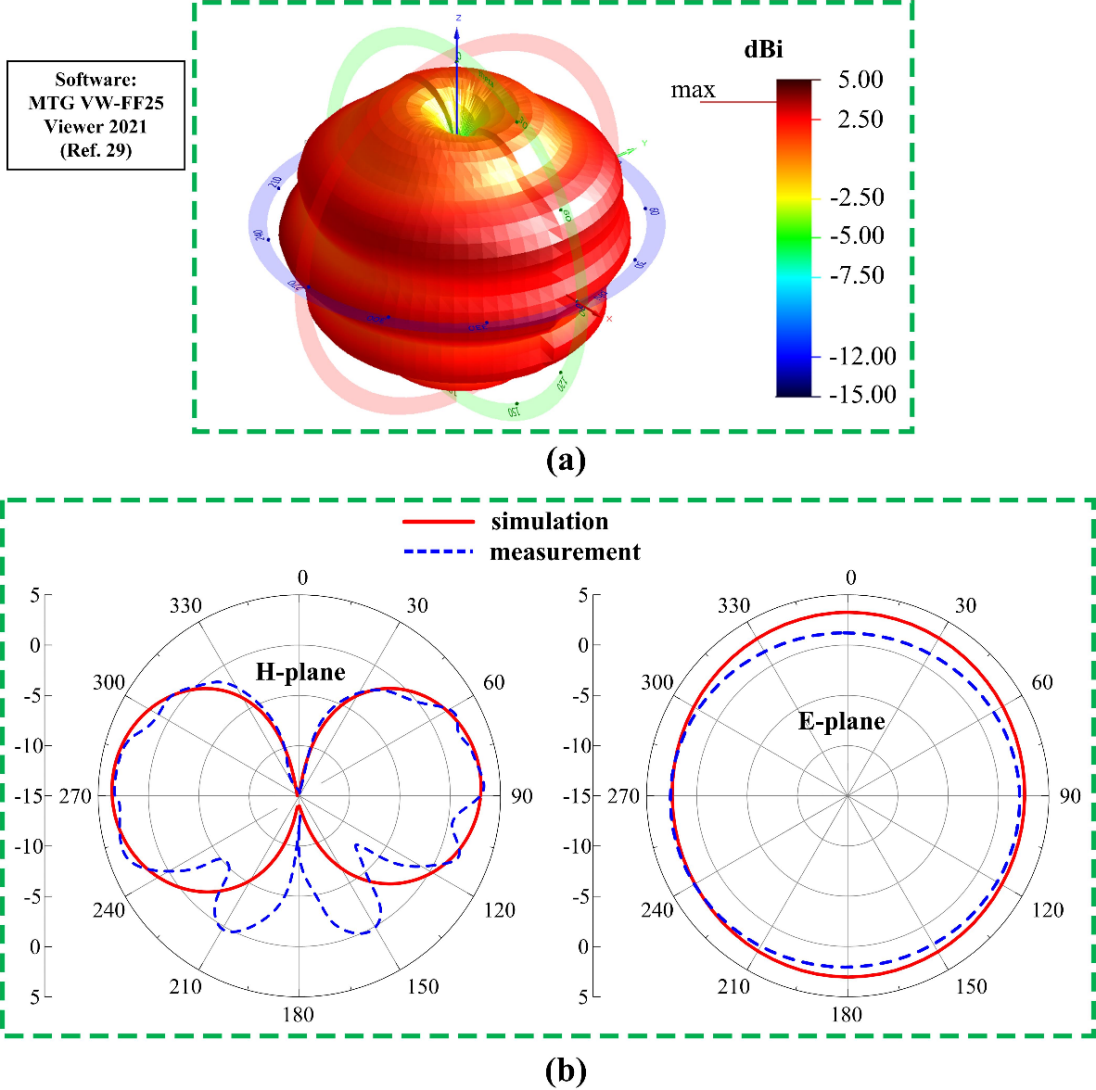
Radiation behavior of Ant- 1 at 0.6 GHz (**a**) measured 3D radiation pattern and (**b**) 1D polar radiation pattern.

To check the suitability of the proposed multi-port antenna sensor, the radiation characteristics of all ports are measured. The radiation behavior characterization setup for Ant- 1 is displayed in Fig. 14. As the antenna will be act as a sensor in actual applications, the proposed antenna is set as an receiver and a standard horn antenna is used as an transmitter in the measurement setup. The positioner rotate the antenna 360° at both E and H plane. It can be seen from the measured antenna radiation pattern performance in Fig. 15 that the Ant- 1 offers a omnidirectional radiation pattern with a gain of 3.7 dBi at 0.6 GHz, respectively. Within the operating band the Ant- 1 shows similar omnidirectional radiation pattern. With this omnidirectional radiation pattern the Ant- 1 can sense signal from all the direction within the operating frequency band between 0.467 GHz and 1.31 GHz.

### Characteristic analysis of the Ant- 2

#### Reflection coefficient

The Ant- 2 of the proposed antenna sensor offers–10 dB impedance bandwidth from 5.63 GHz to 5.96 as shown above in Fig. 13 (b); covering both the 5.8 GHz ISM band and the 5.9 GHz V2X band. The Ant- 2 of the proposed antenna sensor can sense any signal within the offered operating band. Similarly it can communicate with the users by sending data using the V2X and ISM band.

#### Radiation behavior and sensing capabilities of Ant- 2

**Fig. 16:**
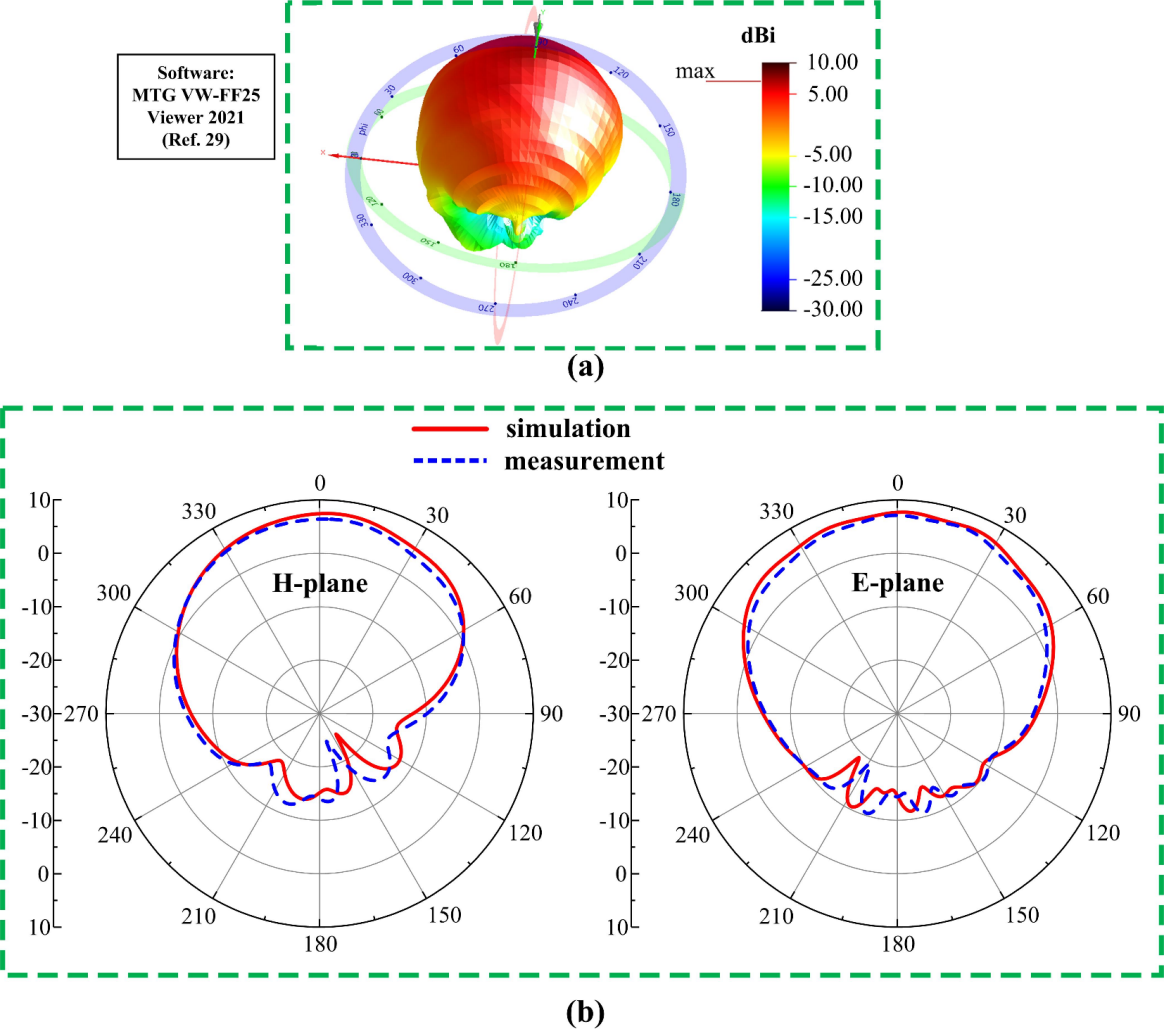
Radiation behavior of Ant- 2 at 5.8 GHz (**a**) measured 3D radiation pattern and (**b**) 1D polar radiation pattern.

The radiation characterization of Ant- 2 is similar to Ant- 1 as shown in Fig. 14 above. The Ant- 2 shows a unidirectional radiation patten. The radiation behavior of Ant- 2 at 5.8 GHz is depicted in Fig. 16. At 5.8 GHz the Ant- 2 offers a HPBW of more than 112.5° with a gain of 6.76 dBi. Within the functional band the Ant- 2 shows similar radiation performance. The wide beam angle and high gain of the Ant- 2 makes it an efficient tool for the 5.8 GHz ISM and 5.9 GHz V2X band sensing and communications.

### Characteristic analysis of the Ant- 3 and Ant- 4

#### Reflection and transmission coefficient

**Fig. 17:**
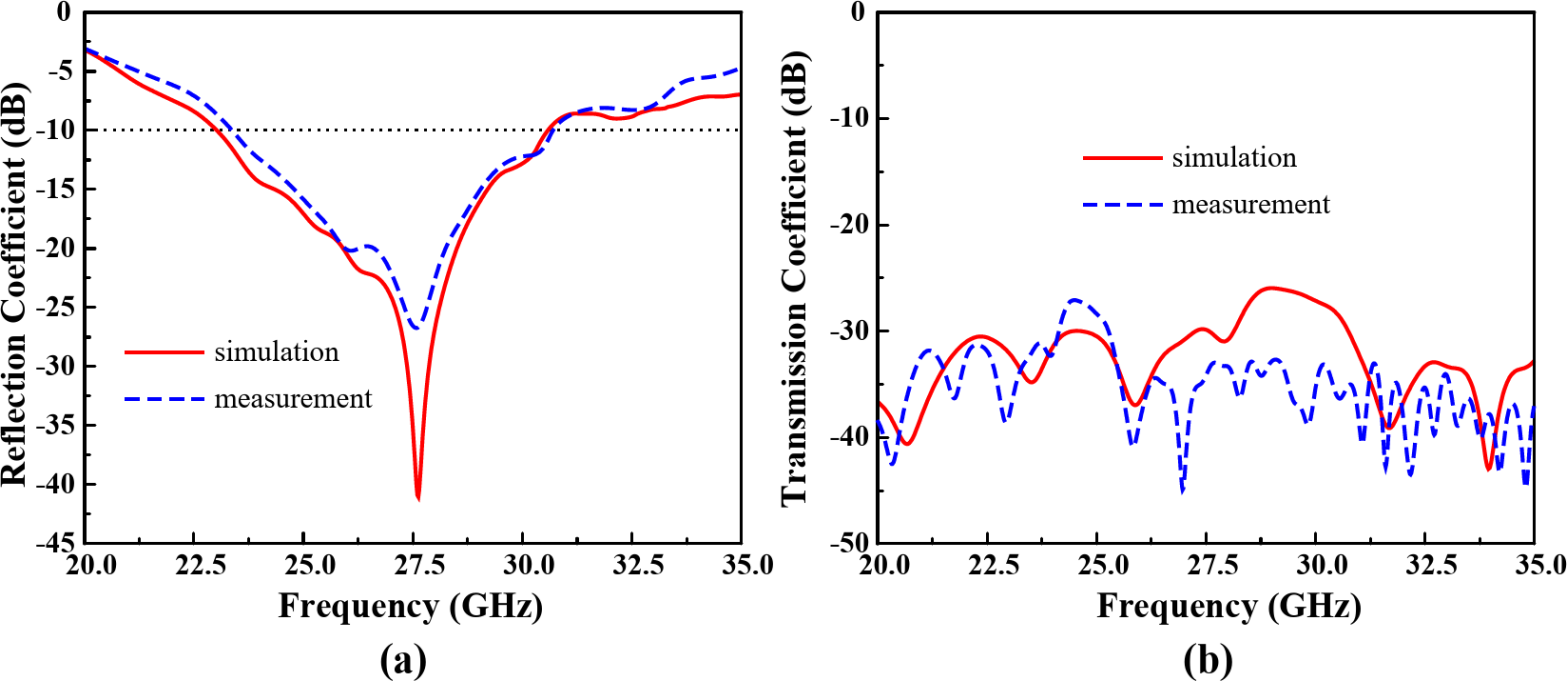
Results of Ant- 3 and Ant- 4 (**a**) reflection coefficient, and (**b**) transmission coefficient.

The 2-port MIMO elements of the proposed antenna sensor which is presented as Ant- 3 and Ant- 4 yields a wide operating bandwidth from 23.02 GHz to 30.37 GHz. The frequency response of Ant- 3 and Ant- 4 is presented in Fig. 17(a). The Ant- 3 and Ant- 4 shows identical frequency response. The simulation and measured results offers a little mismatch due to the connector and cable losses. The offered operating bandwidth of the designed 2-port MIMO system makes it an efficient solution for the 5G millimeter-wave sensing and communications.

The transmission coefficient (*S*_*ij*_ parameters) of the designed 2-port MIMO system at the 5G millimeter-wave band is presented in Fig. 17(b). By utilizing the proposed decoupling structure, parasitic stub with cavity vias, the 2-port MIMO system shows a very low mutual coupling less than − 26.4 dB within the functional bandwidth, which ensure a high element isolation between the MIMO elements. Due to the high isolation, the proposed MIMO system offers excellent MIMO performances.

#### Radiation behavior and sensing capabilities

**Fig. 18:**
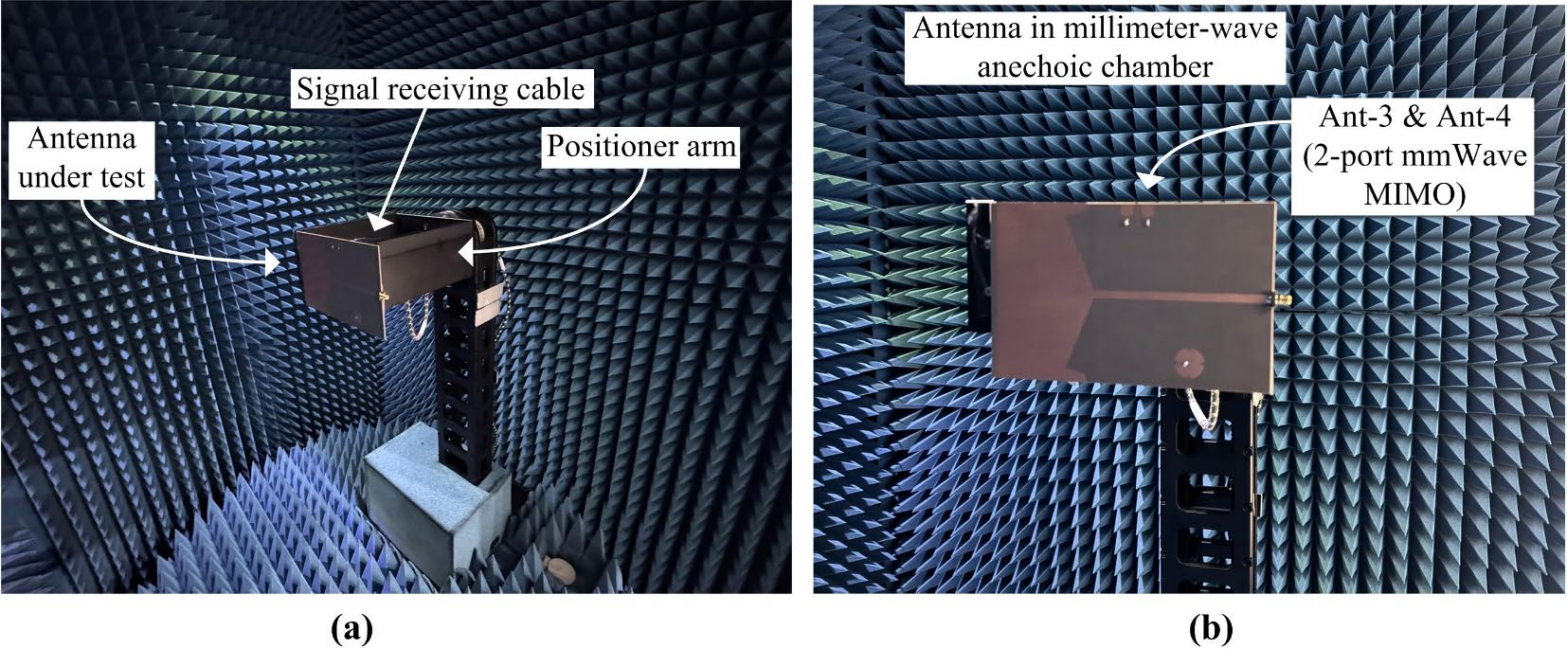
Radiation characterization setup for the designed 2-port MIMO system (Ant- 3 and Ant- 4) in an anechoic chamber (**a**) side view, (**b**) front view.

The radiation characterization setup of the designed 2-port MIMO system for the 5G millimeter-wave band is depicted in Fig. 18. In the measurement setup the proposed antenna sensor act as a receiver whereas the Ant- 3 or Ant- 4 will sense signal transmitted from a standard horn antenna. From the measurement results the suitability of the designed MIMO system can be observed for the sensing and wireless communication.

**Fig. 19:**
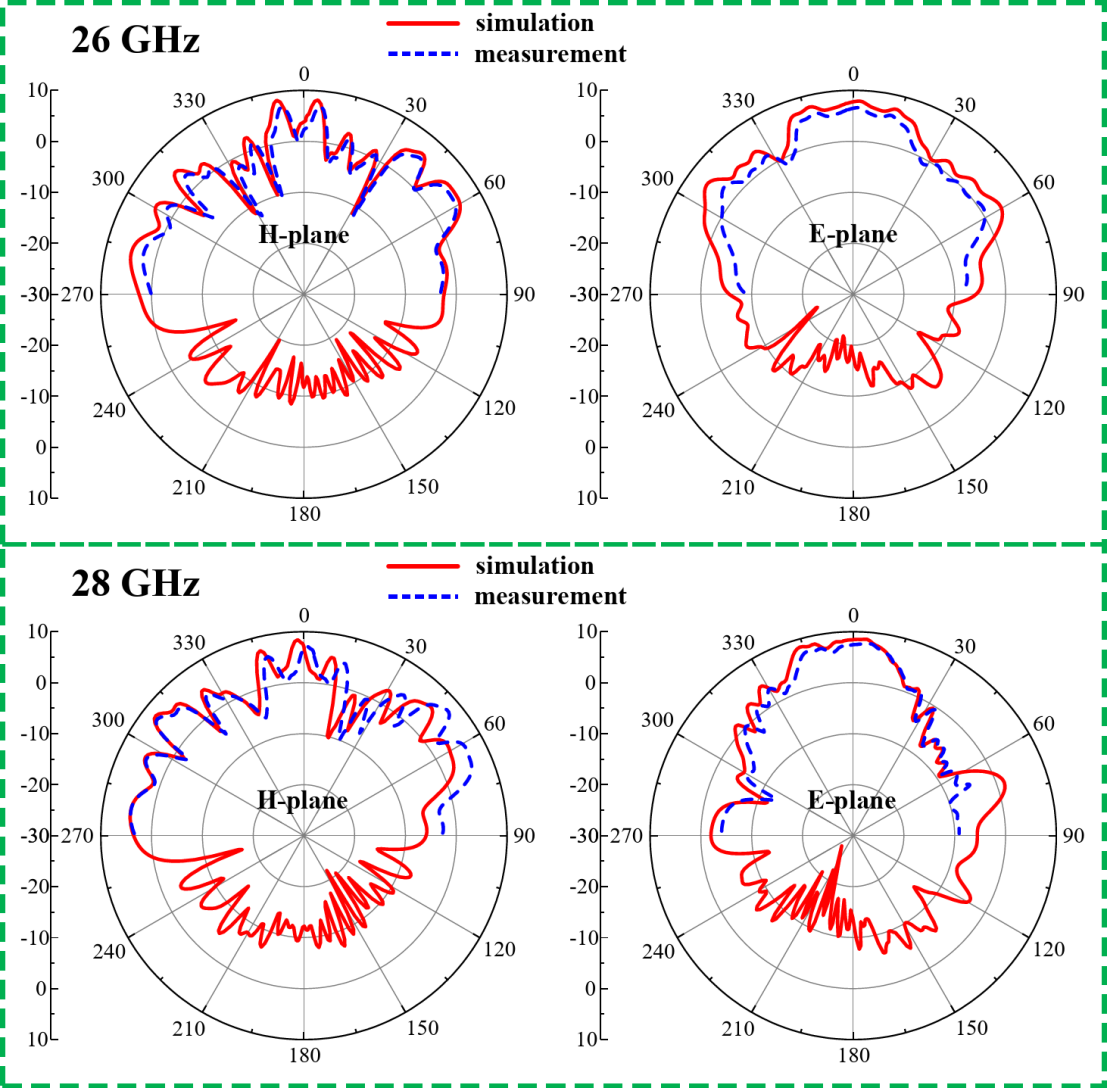
Radiation behavior of Ant- 3 and Ant- 4 at different frequencies.

The radiation behavior of the Ant- 3 and Ant- 4 is presented in Fig. 19. These antennas demonstrate a similar radiation characteristics. These antennas offers a peak gain of 7.68 dBi with an unidirectional radiation. The overall radiation characteristics of Ant- 3 and Ant- 4 allows them to perform efficiently for the 5G millimeter-wave band. Moreover, the simulation result confirms a radiation efficiency of more than 95.6%.

#### MIMO characteristics

**Fig. 20:**
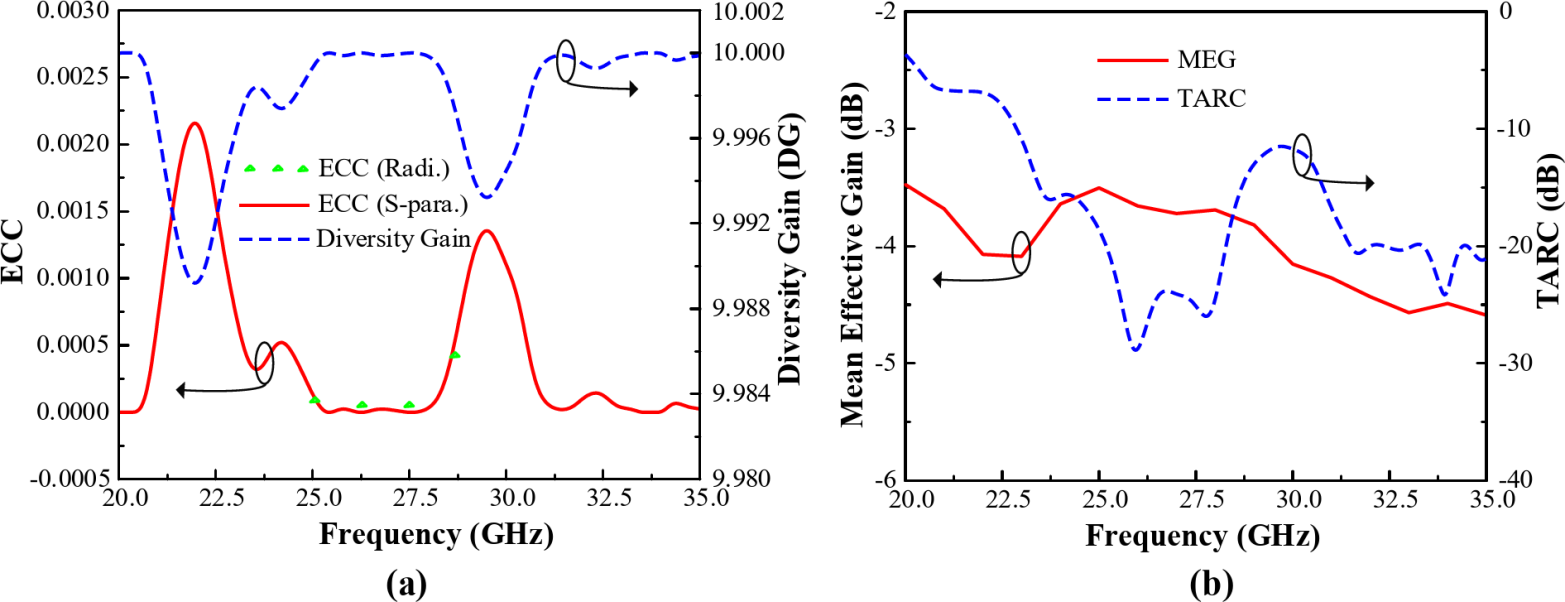
MIMO performances of the designed 2-port MIMO system for 5G millimeter-wave band (**a**) ECC and DG, and (**b**) MEG and TARC.

The diversity performances of the designed 2-port MIMO system are conveyed in Fig. 20. While in Fig. 20(a) contains the envelope correlation coefficient (ECC) and diversity gain (DG). And the mean effective gain (MEG) and total active reflection coefficient (TARC) is presented in Fig. 20(b).

The ECC results of the presented 2-port MIMO system is calculated using Eq. (4), and Eq. (5) from the S-parameters and radiation pattern, respectively^30^. For this 2-port MIMO system *i*,* j*= 1, 2. Excellent element isolation and minimal correlation are guaranteed by a low ECC level, which improves MIMO capabilities^31–34^. The designed MIMO 2-port system shows a ECC level less than 0.0015 within the passband.

4 $$\:{ECC}_{ij}=\frac{{|{S}_{ii}\text{*}{S}_{ij}+{S}_{ji}\text{*}{S}_{jj}|}^{2}}{(1-{\left|{S}_{ii}\right|}^{2}-{{|S}_{ji}|}^{2})(1-{\left|{S}_{jj}\right|}^{2}-{{|S}_{ij}|}^{2})}$$

5 $$\:{ECC}_{ij}=\frac{{\left|{\iint\:}_{0}^{4\pi\:}\left[\overrightarrow{{R}_{i}}\left(\theta\:,\:\phi\:\right)\times\:\overrightarrow{{R}_{j}}\left(\theta\:,\:\phi\:\right)\:\right]d{\Omega\:}\right|}^{2}}{{\iint\:}_{0}^{4\pi\:}{\left|\overrightarrow{{R}_{i}}\left(\theta\:,\:\phi\:\right)\right|}^{2}d{\Omega\:}{\iint\:}_{0}^{4\pi\:}{\left|\overrightarrow{{R}_{j}}\left(\theta\:,\:\phi\:\right)\right|}^{2}d{\Omega\:}\:}$$

The DG demonstrates how the diversity scheme of MIMO elements affects the radiated power, which can be calculated using Eq. (6)^30^. The designed 2-port MIMO system shows an excellent DG performance near to the ideal value of 10 dB.

6 $$\:DG=\:10\sqrt{1-{\left(\text{E}\text{C}\text{C}\right)}^{2}}$$

Form Fig. 20(b) it can be seen than the presented 2-port MIMO system also shows an excellent MEG and TARC performance. The acceptable standard MEG value should be between − 3 and − 12 dB^30^, while the presented 2-port MIMO system yields a MEG value of abound − 3.5 dB. Additionally, it offers a good TARC value of less than − 12 dB within the operating band.

**Fig. 21:**
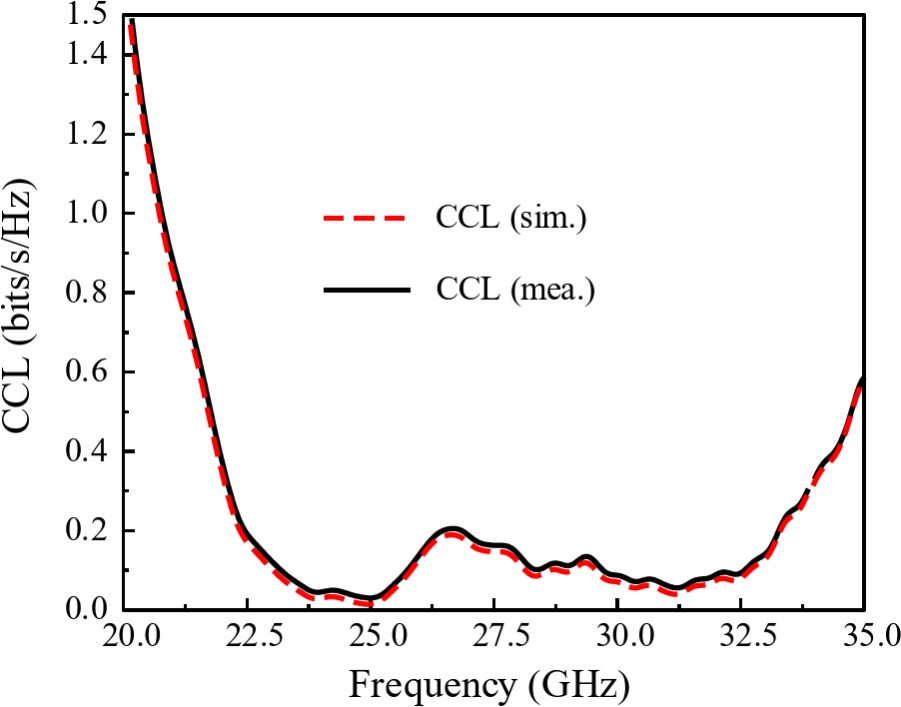
CCL performance of the of the designed 2-port MIMO system for 5G millimeter-wave band.

The channel capacity loss (CCL) of the 2-port millimeter-wave MIMO system is displayed in Fig. 21. The CCL metric quantifies the loss of bits that may occur during transmission^35,36^. The maximum allowable CCL value in any environment is 0.4 bits/s/Hz to ensure the good quality of the communication link^36^. It can be observed from Fig. 21 that the designed 2-port MIMO antenna system for the 5G millimeter-wave communication yields a CCL value less than 0.2 bits/s/Hz within the operating frequency band.

## Performance comparison

Performance comparison of the proposed shared-aperture multi-port antenna sensors antenna with other shared-aperture antennas is presented in Table 2. It can be observed that, only the proposed antenna use a single substrate layer, while all other reported shared aperture antennas required multiple substrate layers. Antennas reported in^21,26^occupy less antenna profile, however they suffer from either complex design procedure^21^or do not offer any band at Sub- 6 GHz^26^. Moreover, none of the reported antennas^20–26^ offers TVWS frequency band for the long-distance communication capabilities specially in rural areas where the number of base stations is limited. Additionally, the proposed antenna offers a very good port isolation of 26.4 dB with a single layer configuration. Overall performance of the proposed share-aperture multi-port antenna sensor, specially the three operating frequency band including the TVWS band makes it a viable solution for the sensing and communication in rural areas.

**Table 2:** Performance comparison with other shared-aperture antennas.

Ref.	Total Profile (mm)	Sub- 6 GHz /5G mmWave	Operating Bands /TVWS Band	Bandwidth (GHz)	Isolation (dB)	Substrate Layers
^20^	3.45	Yes/Yes	Two/No	3.38–3.64 26.4–29.8	40	Quadruple
^21^	0.559	Yes/Yes	Four/No	2.55–2.62 4.74–5.24 26.5–27.4 37.4–38.6	35	Double
^22^	32.54	Yes/Yes	Two/No	5.3–5.49 22.3–26.2	30	Triple
^23^	19.4	Yes/No	Two/No	4–8.6 12–20	25	Double
^24^	> 20	Yes/No	Three/No	1.85–2.15 3.4–3.6 5.4–5.6	15	Triple
^25^	53.2	Yes/No	Two/No	0.77–1.06 2.2–6.6	20	Quadruple
^26^	0.749	No/Yes	Two/No	27.7–33.5 54.1–63.2	30	Quintuple
**Prop.**	**1.524**	**Yes/Yes**	**Three/Yes**	**0.046–1.31** **5.63–5.96** **23.02–30.37**	**26.4**	**Single**

## Conclusion

A shared aperture muti-port antenna sensor is presented for three different operating frequencies covering both Sub- 6 GHz and 5G millimeter-wave bands to accommodate the antenna demand for rural sensing and seamless connectivity. The proposed antenna sensor is created on a 60-mil thick Taconic TLY- 5 substrate having a relative permittivity of 2.2 and loss tangent of 0.0009. The simulated and measured findings show that the proposed antenna sensor can cover TV-white-space (TVWS) frequency band (0.467–1.31 GHz), 5.8 GHz ISM band (5.63–5.96 GHz), and the 5G millimeter-wave frequency band (23.02–30.37 GHz). At the TVWS band the antenna offers a gain of more than 3.14 dBi with an omnidirectional radiation pattern. Deu to the omnidirectional radiation characteristics the proposed antenna at TVWS can act as an antenna sensor which will collect data from all directions. At the 5.8 GHz band the antenna offers a unidirectional radiation pattern with an HPBW of more than 112.5°. The wide beam angle and high gain (6.76 dBi) of the antenna at 5.8 GHz band makes it an efficient tool for the 5.8 GHz ISM and 5.9 GHz V2X band sensing and communications. Additionally, the 2-port MIMO for the 5G millimeter-wave sensing and communications also offers excellent MIMO diversity performances with a gain of 7.68 dBi. Overall performance of the proposed share-aperture multi-port antenna sensor, especially the three operating frequency band including the TVWS band makes it a viable solution for the sensing and communication in rural areas.

## Data Availability

The presented paper contains all the data required for evaluating the findings of this research. The corresponding author can be contacted for more data regarding this work.

## References

[1] Xiao, Y. et al. Multiband and low-specific-absorption-rate wearable antenna with low profile based on highly conductive graphene assembled film. *IEEE Antennas Wirel. Propag. Lett.* **22**, 2195–2199 (2023).

[2] Hasan, M. M., Rahman, M., Faruque, M. R. I., Islam, M. T. & Khandaker, M. U. Electrically compact SRR-loaded metamaterial inspired quad band antenna for Bluetooth/WiFi/WLAN/WiMAX system. *Electronics* **8**, 790 (2019).

[3] Ullah, N. et al. Design and development of a compact, wide-angle metamaterial electromagnetic energy harvester with multiband functionality and polarization-insensitive features. *Sci. Rep.* **14**, 19000 (2024). 39152247 10.1038/s41598-024-69976-2 PMC11329642

[4] Siddiqui, S. I. et al. A dual-band high-gain beam steering antenna array for 5G sub-6 ghz base station. *Sci. Rep.* **14**, 26517 (2024). 39489742 10.1038/s41598-024-75822-2 PMC11532557

[5] Sufian, M. A. et al. High gain quasi-omnidirectional dipole array fed by radial power divider for millimeter-wave IoT sensing. *Sci. Rep.* **14**, 16279 (2024). 39009638 10.1038/s41598-024-67032-7 PMC11251259

[6] Singh, A. K. & Tripathy, M. R. M-shaped ultra-wideband monopole antenna for TVWS CPE. *Proc. 5th Int. Conf. Signal Process. Integr. Netw. (SPIN)*, 240–243 (2018).

[7] Kumar, S., Rayavarapu, N. & Naidu, P. V. A novel frequency reconfigurable antenna for smart grid applications in TV white space band. *Iran. J. Electr. Comput. Eng.* **13**, 611–618 (2023).

[8] Ma, Y. System embedded antenna for portable TVWS cognitive radio transceiver. *IEEE Antennas Wirel. Propag. Lett.* **14**, 265–268 (2015).

[9] Meghwal, A., Saini, G. spsampsps Dhaliwal, B. S. A unique multident wideband antenna for TV white space communication. *Proc. Int. Conf. Paradigms Comput. Commun. Data Sci. (PCCDS)*, 745–756 (2023).

[10] Zalki, G. & Bakhar, M. Microstrip patch antenna array inspired by split ring resonators for 5.8 ghz applications. *Wirel. Pers. Commun.* **138**, 321–331 (2024).

[11] Yu, Y., Tong, Y., Shunqin, X., Rodríguez-Piñeiro, J. & Yin, X. Statistical channel model based on passive measurements for indoor WiFi communications at 2.4 ghz and 5.8 ghz bands. *IEEE Antennas Wirel. Propag. Lett.* **23**, 778–782 (2024).

[12] Chen, Q., Cheng, Y. F., Peng, Q. H. & Liao, C. A wideband and compact millimeter-wave antenna array fed by printed ridge gap waveguide for 5G applications. *J. Electromagn. Eng. Sci.* **24**, 485–493 (2024).

[13] Wang, J., Cao, Y., Che, W. & Xue, Q. Wide-angle beam-scanning millimeter-wave antenna array using phase-controlled beam-steerable elements. *IEEE Trans. Antennas Propag.* **72**, 3730–3735 (2024).

[14] Kareem, F. R., Ibrahim, A. A. & Abdalla, M. A. Triple-band monopole textile wearable antenna for IoMT application. *IEEE Sens. J.* **23**, 23377–23387 (2023).

[15] An, W., Han, K., Luo, Y. & Wang, Y. Low-profile lightweight slot array with solar cells for 5.8 ghz band. *IEEE Antennas Wirel. Propag. Lett.* **22**, 1476–1480 (2023).

[16] Fakhriddinovich, A. U., Sufian, M. A., Awan, W. A., Hussain, N. & Kim, N. A compact antenna with multiple stubs for ISM, 5G sub-6-GHz, and WLAN. *IEEE Access.* **11**, 130418–130425 (2023).

[17] Tan, Q. et al. A circularly polarized magneto-electric dipole antenna array with wide AR and impedance bandwidth for millimeter-wave applications. *IEEE Antennas Wirel. Propag. Lett.* **22**, 2250–2254 (2023).

[18] Ullah, U., Koziel, S., Pietrenko-Dabrowska, A. & Kamal, S. A planar-structured circularly polarized single-layer MIMO antenna for wideband millimetre-wave applications. *Eng. Sci. Technol. Int. J.* **57**, 101819 (2024). 10.1038/s41598-024-60678-3 PMC11065865 38698012

[19] Dwivedi, A. K. et al. A Taguchi neural network–based optimization of a dual-port, dual-band MIMO antenna encompassing the 28/34 ghz millimeter wave regime. *Sci. Rep.* **15**, 6026 (2025). 39972019 10.1038/s41598-025-90103-2 PMC11839919

[20] Ding, X. H. et al. A dual-band shared-aperture antenna for microwave and millimeter-wave applications in 5G wireless communication. *IEEE Trans. Antennas Propag.* **70**, 12299–12304 (2022).

[21] Ni, S., Li, X., Yin, J., Wang, Q. & Zhang, J. A compact quad-band shared-aperture antenna based on electromagnetic transparent structure. *IEEE Antennas Wirel. Propag. Lett.* **21**, 1020–1024 (2024).

[22] Liu, Z. G., Yin, R. J. & Lu, W. B. A novel dual-band shared-aperture antenna based on folded reflectarray and Fabry–Pérot cavity. *IEEE Trans. Antennas Propag.* **70**, 11177–11182 (2022).

[23] Li, M. et al. A broadband dual-polarized C-/Ku-band shared aperture antenna array for satellite communications. *IEEE Trans. Antennas Propag.* **72**, 8840–8845 (2024).

[24] Sun, Y. et al. Tri-band dual-polarized shared-aperture antenna arrays with wide-angle scanning and low profile for 5G base stations. *IEEE Trans. Antennas Propag.* **72**, 2455–2467 (2024).

[25] Zhang, D. & Wu, Q. A shared-aperture antenna with high aperture reuse efficiency and high isolation based on the quasi-Yagi structure. *IEEE Trans. Antennas Propag.* **71**, 8504–8513 (2023).

[26] Wang, X., Xiao, G., Cheng, H. & Zhao, B. An orthogonally polarized shared-aperture antenna based on structure reuse of metasurface. *IEEE Antennas Wirel. Propag. Lett.* **22**, 2821–2825 (2023).

[27] CST 2023 Academic Software. Software license provider: https://www.emagtech.co.kr/

[28] Balanis, C. A. *Antenna Theory: Analysis and Design* 4th edn (Wiley, 2016).

[29] EMTI-Antenna Measurement Facility. Seoul, South Korea. Homepage. Accessed: Nov. 19, 2024. [Online]. Available: http://emti.or.kr/

[30] Sufian, M. A., Hussain, N. & Kim, N. Quasi-binomial series-fed array for performance improvement of millimeter-wave antenna for 5G MIMO applications. *Eng. Sci. Technol. Int. J.* **47**, 101548 (2023).

[31] Sharma, M. et al. Flexible four-port MIMO antenna for 5G NR-FR2 tri-band MmWave application with SAR analysis. *Sci. Rep.* **14**, 29100 (2024). 39582070 10.1038/s41598-024-79859-1 PMC11586418

[32] Zheng, X. et al. A low-coupling broadband MIMO array antenna design for Ku-band based on metamaterials. *J. Electromagn. Eng. Sci.* **24**, 666–673 (2024).

[33] Haque, M. A. et al. Multiband THz MIMO antenna with regression machine learning techniques for isolation prediction in IoT applications. *Sci. Rep.* **15**, 7701 (2025). 40044756 10.1038/s41598-025-89962-6 PMC11882809

[34] Addepalli, T. Effective area reduction & surface waves suppression of a novel four-element MIMO antenna exclusively designed for dual band 5G sub 6 ghz (N77/N78 & N79) applications. *Wirel. Netw.* **31**, 1463–1479 (2025).

[35] Sufian, M. A. et al. Mutual coupling reduction of a circularly polarized MIMO antenna using parasitic elements and DGS for V2X communications. *IEEE Access.* **10**, 56388–56400 (2022).

[36] Addepalli, T. & Compact MIMO diversity antenna for 5G Sub-6 ghz (N77/N78 and N79) and WLAN (Wi-Fi 5 and Wi-Fi 6) band applications. *Wirel. Pers. Commun.* **132**, 2203–2223 (2023).

